# Work-related coping behaviour and experience patterns in university students: a review of 20  years of research

**DOI:** 10.3389/fpsyg.2023.1062749

**Published:** 2023-04-18

**Authors:** Ivana Mašková

**Affiliations:** Department of Psychology, Faculty of Education, University of South Bohemia, České Budějovice, Czechia

**Keywords:** burnout vulnerability, coping behaviour, occupational stress, university students, work-related patterns

## Abstract

Work-related coping behaviour and experience patterns (WCEP) is the conceptualisation of personal experience with occupational stress and of the typical behavioural responses for coping with such stress. The objective of this review, which is based on 69 references that used the WCEP inventory in university students, is to offer a comprehensive overview of the findings on WCEP and their correlates in the student population. The results of the published studies consistently show that female students, teacher education students (compared to medical students) and students who receive insufficient social and financial support are at greater risk for being assigned to work-related patterns that indicate vulnerability to burnout and occupational health issues. Moreover, students assigned to these patterns, especially to the resigned (burnout) pattern, are prone to manifest other negative characteristics, such as less adaptive personality traits and coping strategies, vulnerability to stress, lower quality motivation, lack of commitment to the chosen career and suitability for the profession, and impaired physical and mental health. In contrast, the most desirable correlates, such as adaptive personality traits, higher quality motivation, commitment to the chosen career, suitability for the profession, stress resistance, adaptive coping and better physical and mental health, were related to the healthy ambitious pattern. Nevertheless, further research is needed to analyse work-related coping behaviour and experience patterns beyond the German speaking population to increase the generalisability of the findings.

## 1. Introduction

Among various occupational and age groups, university students have consistently been shown to be at risk of higher distress, anxiety, depression and poor mental health outcomes in general (e.g. Stallman, 2010; Evans et al., 2018; Rehman et al., 2021). Such issues may arise in part from the upheavals of the emerging adult life stage (lasting from 18 to about 29 years) that largely overlaps with the phase of university studies (Arnett and Schwab, 2012). In this respect, the characteristics of emerging adulthood, i.e., identity explorations, instability, self-focus, feeling in-between and possibilities/optimism, have specific implications for mental health. Specifically, identity struggles, frequent changes accompanied by lack of social support or feeling of not reaching adulthood yet can trigger anxiety and depression in emerging adults (Arnett et al., 2014). On the other hand, as suggested by Nelson et al. (2008), the above-mentioned characteristics differentiating emerging adulthood from other life stages make emerging adulthood a particularly important time for establishing and intervening on long-term (health) behaviour patterns, including positive coping behaviours to deal with occupational demands that could, in turn, foster emerging adults’ mental health and well-being. Therefore, the unique phase of emerging adulthood/higher education is of primary interest for the present study, which focuses on patterns of dealing with occupational demands and their various correlates. More specifically, a conceptualisation of personal experience with occupational stress and the typical behavioural responses used in coping with such stress referred to as *work-related coping behaviour and experience patterns* (*Arbeitsbezogenes Verhaltens- und Erlebensmuster* in its original German version) (Kieschke and Schaarschmidt, 2008) is brought into spotlight.

## 2. Work-related coping behaviour and experience patterns

Work-related coping behaviour and experience patterns (WCEP) can be gaged by the same named inventory involving 11 dimensions grouped into three main areas: professional commitment, coping capacity and subjective well-being (Kieschke and Schaarschmidt, 2008; Schaarschmidt and Fischer, 2008). Four profiles or patterns representing an individual’s capability to deal with professional demands were identified by a cluster analysis of the 11 dimensions. Assigning an individual to one of the distinct patterns (G, S, A, and B) based on the highest match between the individual scores and the four patterns provides information about individual work-related health risks and motivational deficits (Schaarschmidt and Kieschke, 2007; Kieschke and Schaarschmidt, 2008; Schaarschmidt and Fischer, 2008). The characteristics of these patterns are presented in Table 1.

**Table 1:** Description of the four work-related patterns, their characteristics and specifics.

Pattern label	Pattern description	Pattern characteristics			Pattern-related specifics and health risks
		Professional commitment	Coping capacity	Subjective well-being	
G	The healthy ambitious pattern	High	High	High	health promoting attitude to work, optimal professional motivation, no health risks
S	The unambitious pattern	Very low	High	High average	greatly reduced professional motivation (restriction of efforts at work to only what is absolutely necessary), no health risks
A	The excessively ambitious risk pattern	Excessively high	Low	Low average	resemblance to type-A behaviour (workaholism), over-motivation, vulnerability to health risks, especially to cardiovascular diseases
B	The resigned risk pattern	Low	Low	Low	resemblance to the symptomatology of the late stages of the burnout syndrome, reduced professional motivation, vulnerability to burnout, vulnerability to health risks, especially to psychosomatic diseases

G refers to ‘good health’, S refers to ‘sparing personal investment at work’, A refers to ‘ambitious’, B refers to ‘burnout’. Information based on Kieschke and Schaarschmidt (2008), Schaarschmidt and Fischer (2008), and Schaarschmidt and Kieschke (2007).

These profiles represent a relatively consistent style of dealing with professional demands. Nevertheless, if a spontaneous pattern transition occurs in the long-term, it is likely to be a transition toward less desirable patterns (Kieschke and Schaarschmidt, 2008). Thus, the main aim of pattern assessment is the early identification of vulnerable individuals that can lead to timely psychological interventions to correct the undesirable patterns or prevent such vulnerable individuals from entering highly demanding professions (Künsting et al., 2012). The importance of the early recognition of vulnerable individuals is reflected in a substantial proportion of WCEP research that focuses specifically on individuals in their earliest career stage–university students.

To facilitate the recognition of vulnerable individuals, student focused WCEP studies frequently aim to identify WCEP correlates that can function as protective or risk factors of (un)desirable work-related patterns. The objective of this review is to summarize the existing findings on WCEP and their correlates in the student population available from the year 2002 when the first study on WCEP in students was published to the year 2022 when the present review was conducted. The review is based on 69 references that used the WCEP inventory in university students and provide information on the WCEP distribution and/or related correlates. For a comprehensive outline of the literature, see the Supplementary material.

## 3. Review methodology

### 3.1. Eligibility criteria

The eligibility criteria for this review covered all the published empirical literature (journal articles, books, reports, theses and conference outputs) that used the WCEP inventory in university students and presented information on the distribution of the four patterns according to Schaarschmidt and Fischer (2008) and/or their correlates. Only records in English and German were included. The records that focused on other populations than undergraduate university students (or merged data of students with that of other populations) were excluded, as well as those involving teacher trainees in practical training, known as the second phase of teacher education (‘Lehramtsreferendariat’). Excluded were also records where the participants were not assigned to one of the four patterns (the patterns were not calculated), the calculation did not correspond to the original typology by Kieschke and Schaarschmidt (2008), or the presentation of the WCEP distribution was unclear.

### 3.2. Search strategy

Searched were all keywords relevant to WCEP combined with the focus on students (search string: (Arbeitsbezogenes Verhaltens- und Erlebensmuster OR AVEM OR Work-related coping behaviour and experience patterns) AND (student)). The search strategy targeted the Web of Science and Scopus databases and the search engines Google and Google Scholar, supplemented with a search in the ResearchGate database and contact with prominent authors in the field of WCEP. Finally, reference lists of the identified articles were screened manually. The searches were performed between June 1st and September 15th 2022.

### 3.3. Literature selection

**Figure 1:**
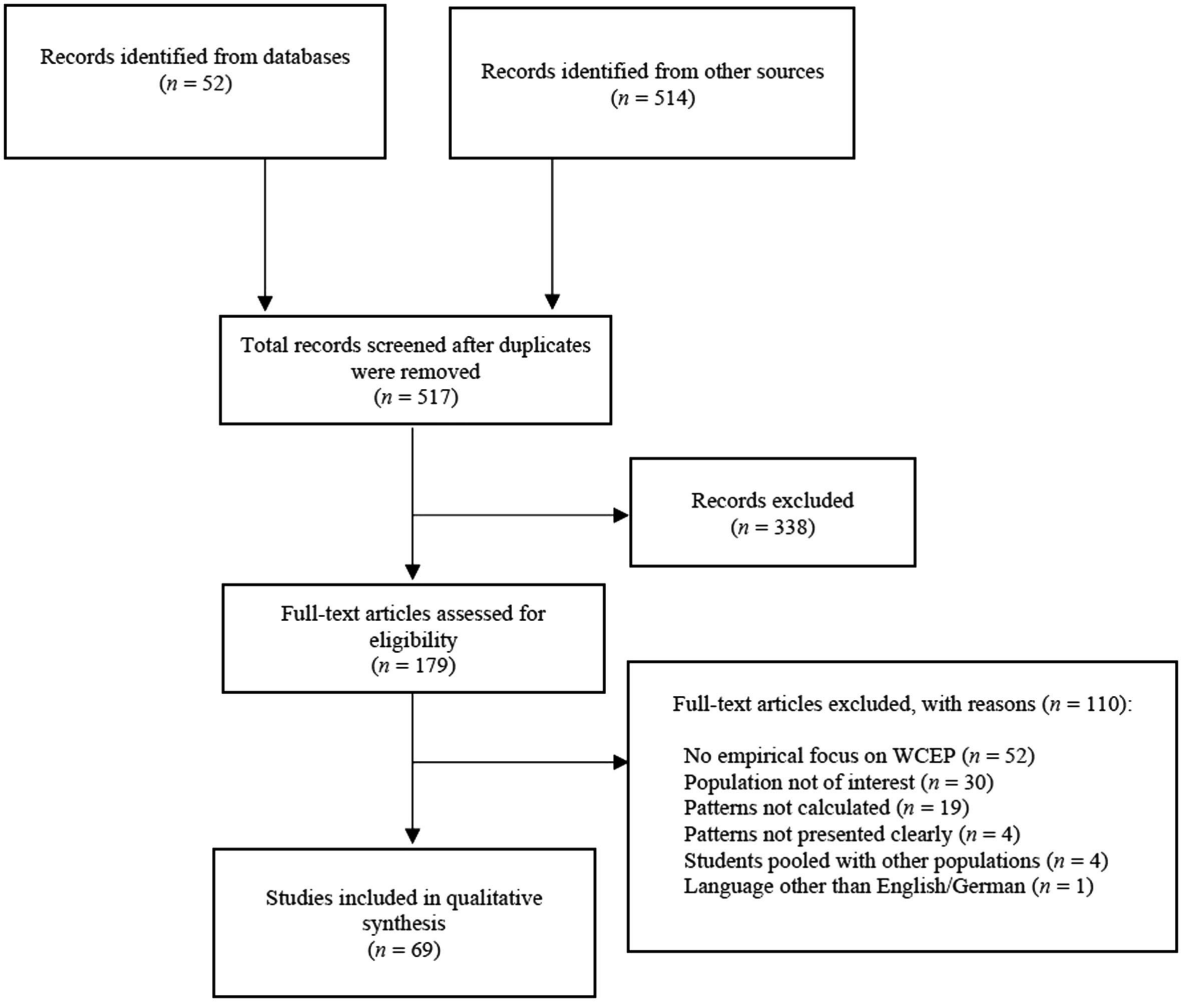
A flowchart of the search strategy. Flowchart adapted from PRISMA 2020 flow diagram for new systematic reviews which included searches of databases, registers and other sources available at https://prisma-statement.org/PRISMAStatement/FlowDiagram.

The initial search yielded 566 sources, including 30 from Web of Science, 22 from Scopus and 514 from additional searches. After removing 49 duplicates, the titles and abstracts of 517 records were screened resulting in the exclusion of 338 records that did not meet the eligibility criteria. After reading the remaining 179 full texts, the author decided to include 69 records (see Figure 1). Data extracted from the sources include a reference, the country of data origin and the higher education institution of data collection if available, the period of data collection, sample characteristics, the percentual distribution of WCEP in the sample and top-line findings on WCEP correlates (see Supplementary material).

### 3.4. Quality assessment

The quality of the included studies was assessed using the Mixed Methods Appraisal Tool (MMAT), which is a critical appraisal tool designed to appraise the methodological quality of qualitative, quantitative and mixed method studies (Hong et al., 2018). Studies that relied on the same dataset were assessed as one unit since their methodological approach was shared to a large extent. For the purpose of the critical appraisal, all relevant information provided across studies within a unit was considered. Out of the 54 assessed units, one was classified as a quantitative randomized controlled trial (Schaefer, 2012; Çelebi et al., 2014), one as a quantitative non-randomized study (Wild et al., 2014), one as a mixed method study (Cramer, 2012) and the remaining 51 units were quantitative descriptive studies. Units were assessed according to five criteria relevant to the respective category (see Hong et al., 2018). A score was assigned for each of the criteria based on whether the criterion was met: 2 = ‘yes’; 1 = ‘partially’; 0 = ‘no/cannot tell’. Thus, the final quality assessment score for each unit could range from 0 to 10. Following current research practices, the methodological quality of each unit was classified as poor (final score ≤ 5), moderate (final score 6–7) or good (final score ≥ 8) (Squires et al., 2011; Li et al., 2015; Kamal et al., 2021). As a result, 26 units were classified as good quality, 11 as moderate quality and 10 as poor quality (see Supplementary material). Seven units were not assessed, as the records provided little information on the study methodology due to the nature of the record (e.g., conference posters).

## 4. WCEP and their correlates in university students–what we know so far

The most researched group within the student-focused WCEP research framework are teacher education students (e.g., Bauer, 2019; Mašková et al., 2022) followed by medical students (e.g., Kötter et al., 2021; Afshar et al., 2022). There is also evidence of WCEP distribution in psychology (Nowik and Franke, 2009; Reichl et al., 2014; Meiseneder, 2015), nursing (Kada, 2014), dentistry (Cramer, 2012), law (Römer et al., 2012, 2013), business/economics (Nowik and Franke, 2009; Jäger, 2017), STEM (Voltmer et al., 2019), natural science (Obst and Kötter, 2020), music (Nusseck and Spahn, 2013), sport (Fischer et al., 2018) and theology students (Voltmer et al., 2011b). The vast majority of WCEP research studies focusing on university students was conducted in Germany (e.g. Reichl et al., 2014; Afshar et al., 2022) while a small number of studies was also conducted in other German-speaking countries: Austria (e.g. Beer and Benieschek, 2012; Lüftenegger et al., 2019) and Switzerland (Albisser and Kirchhoff, 2007; Deiglmayr et al., 2018). Additionally, one study involved teacher education students from the Czech Republic (Mašková et al., 2022).

The following sections provide a review of the findings on WCEP correlates identified in student focused WCEP research.

### 4.1. Gender

The WCEP distribution tends to be affected by gender. Generally, women in all study fields were more likely than men to be assigned to the risk patterns, especially pattern A (Voltmer et al., 2010a; Rothland, 2011; Meier, 2015; Fischer et al., 2018; Afshar et al., 2022). In contrast, men were more likely to be assigned to pattern S (Rothland, 2011; Martin, 2012; Römer et al., 2013; Mašková et al., 2022) except for fifth-year medical students and Swiss teacher education students where women were more likely to be assigned to pattern S (Voltmer et al., 2008; Deiglmayr et al., 2018).

### 4.2. Study field

Since most studies focused on teacher education and medical students, comparing the WCEP distribution in students from various study fields was less straightforward. Specifically in teacher education students, most studies revealed that the distribution of WCEP was unaffected by teaching specializations (Künsting et al., 2012; Martin, 2012; Römer et al., 2013; Würfl, 2013; Meier, 2015; Mašková et al., 2022), although Boxhofer (2013) and Cramer (2012) found a tendency for teacher education students focused on special schools to be assigned to the pattern S. Compared to teacher education, the prevalence of risk patterns seemed to be higher in dentistry, sport, music, natural sciences, psychology and law students (Cramer, 2012; Nusseck and Spahn, 2013; Römer et al., 2013; Reichl et al., 2014; Fischer et al., 2018; Obst and Kötter, 2020). In contrast, the majority of studies showed that the prevalence of risk patterns tends to be lower in medical students (e.g., Aster-Schenck et al., 2010; Voltmer et al., 2010a), although there is also evidence on increased likelihood of pattern A in medical students at the beginning of medical education (Afshar et al., 2022). Specifically, teacher education students displayed an increased prevalence of the S pattern compared to dentistry (Cramer, 2012), sport (Fischer et al., 2018), law (Römer et al., 2013) or music students (Nusseck and Spahn, 2013), but a decreased prevalence of the S pattern compared to medical students (especially in the later phases of medical education) (e.g. Voltmer et al., 2010a, 2012).

### 4.3. Study phase

Findings related to the WCEP distribution in the initial and more advanced phases of higher education are rather inconsistent across the various fields of study. In teacher education students, cross-sectional studies showed either no difference in WCEP distribution in the various study phases (Buss, 2002; Römer et al., 2013; Bauer, 2019) or a higher prevalence of the risk patterns in the more advanced study phases (Schröder and Kieschke, 2006; Rothland, 2011). The latter was also observed in law and STEM students (Römer et al., 2013; Voltmer et al., 2019). Presenting a different trend in teacher education students, Grözinger and Förster (2016) found a slight increase of pattern S in fourth-year students compared to first-year students within a longitudinal framework. A similar but more pronounced trend was repeatedly observed in medical students who displayed a steady increase in the unambitious pattern S at the expense of the healthy pattern G from the initial to the final phases of their studies (Aster-Schenck et al., 2010; Voltmer et al., 2021a; Afshar et al., 2022). In sum, the WCEP distribution tended to either persist or transition toward less desirable patterns during the course of study.

### 4.4. Background characteristics

Student background characteristics seem to have only a limited impact on the WCEP distribution in general. Whereas Jäger (2017) found that younger students were more likely to be assigned to pattern G than older students, other studies did not confirm the link between age and WCEP (Buss, 2002; Aster-Schenck et al., 2010; Cramer, 2012; Meier, 2015). Likewise, neither marital/relationship status nor children were correlated with work-related patterns (Aster-Schenck et al., 2010; Jäger, 2017; Afshar et al., 2022). On the other hand, the likelihood of being assigned to a healthy pattern was higher for students who had a personally important job while studying (Mašková et al., 2022) but who were not employed full-time (Jäger, 2017). Further, the WCEP distribution was more favorable in students who received financial support, whose parents had higher socioeconomic standing (Cramer, 2012; Rumpler, 2013; Afshar et al., 2022) and who had higher social support (Voltmer et al., 2008; Hamdan, 2012; Jäger, 2017).

### 4.5. Learning and academic achievement

In terms of student learning and achievement, the most notable differences tend to exist between the under-motivated S types followed by B types on the one hand and the over-motivated A types followed by the healthy motivated G types on the other hand. In this respect, S-type teacher education students recorded the lowest number of hours per week devoted to studying and the lowest level of increase in basic pedagogical knowledge, while A types recorded the highest in both cases. The findings related to both B and G types were relatively inconclusive (Rumpler, 2013; Römer et al., 2017). Further, S- and B-type students had a less pronounced learning goal orientation and showed a less intensive use of learning strategies compared to G and A types (Künsting et al., 2012), who also had the highest level of self-perceived academic achievement (Aster-Schenck et al., 2010; Voltmer et al., 2012). Nevertheless, although both medical and teacher education G- and A-type students tended to achieve better academic results (Künsting et al., 2012; Voltmer et al., 2012), the differences among the four types were statistically insignificant in terms of the final high school grade (Aster-Schenck et al., 2010), university grades (Künsting et al., 2012; Voltmer et al., 2012) or the number of exams passed (Künsting et al., 2012).

### 4.6. Personality factors

In terms of personality, students assigned to the risk patterns repeatedly displayed less favorable outcomes compared to those assigned to the unambitious pattern or to the healthy pattern. Specifically, A–and B-type individuals displayed higher levels of neuroticism than G and S types; reversely, extraversion was more prominent in G-type individuals than in those assigned to the less desirable patterns. Conscientiousness, on the other hand, was scored high in both G–and A-type students, and low in B and S types (Cramer, 2012; Künsting et al., 2012; Reichl et al., 2014; Römer et al., 2017; Lüftenegger et al., 2019). Further, G- and S-type students were more resilient and displayed higher levels of mindfulness and self-efficacy than A and B types, who, on the other hand, tended to be more pessimistic, irritable and less tolerant of uncertainty than their G–and S-type counterparts (Dietrich et al., 2015; Meiseneder, 2015; Awenius, 2019; Bauer, 2019). Finally, G-type theology students displayed higher levels of spirituality which protected them from the burnout pattern (Voltmer et al., 2011b).

### 4.7. Study and career choice motivation

A review of the research on the interrelatedness of WCEP and motivation in teacher education students is provided by Mašková et al. (2022). In sum, problematic or low-quality motives are usually related to the B pattern, while high quality forms of motivation are found in G types. In several studies, the motivational profile of S and A types resembles that of the B and G types, respectively (e.g., Künsting et al., 2012; Rothland, 2012; Reichl et al., 2014; Mašková et al., 2022).

### 4.8. Commitment to students’ career choice

Students’ motivation is closely interconnected with commitment to their career choice. Evidence has consistently shown that B-type teacher education students were the least satisfied with their studies and their choice of a teaching career (Albisser and Kirchhoff, 2007; Rothland, 2011; Künsting et al., 2012), whereas the most satisfied were typically the G (Rothland, 2011; Rumpler, 2013) and A types (Künsting et al., 2012). Similarly, G- and A-type medical and natural science students displayed the highest identification with their studies while B types showed the lowest (Obst et al., 2017; Obst and Kötter, 2020). Likewise, students with a high subjective certainty about their choice of a teaching career and with the intention of pursuing such a career after completing their studies displayed the most desirable WCEP profiles; the opposite applied to students who were uncertain or did not intend to become teachers (Schaarschmidt, 2005; Rothland, 2011). However, conflicting evidence exists on the time point of the decision to pursue a teaching career. Whereas in German students an earlier decision increased the probability of being assigned to the G pattern (Rothland, 2011), in Austrian students the life-anchored decision was linked to the higher likelihood of being assigned to the risk pattern A; in contrast, the most favorable WCEP profiles typically showed those who decided to pursue a teaching career later after entering teacher education (Lüftenegger et al., 2019). Further, Cramer (2012) found that the highest likelihood of being assigned to the pattern B showed students who set themselves against the teaching style they experienced as pupils and seek to change and modernize the educational system. Finally, although previous completed studies did not affect the WCEP distribution (Afshar et al., 2022), teacher education students, who had not completed their previous studies or were enrolled in multiple degree courses concurrently (Mašková et al., 2022), and male students, who had chosen teacher education as a second choice, were more likely to be assigned to pattern B (Rothland, 2011).

### 4.9. Suitability for the future profession

Adding to the above-mentioned evidence which shows that B-type students lack commitment to their career choice, several studies also suggest that B types, in contrast to G types, may be unsuitable for their future profession. Specifically, B-type teacher education students tend to lack beliefs, expectations, interests and competencies necessary for the teaching profession (Albisser and Kirchhoff, 2007; Kaub et al., 2014; Meier, 2015; Deiglmayr et al., 2018). Moreover, in medical students, patterns B and A were linked to lowered levels of empathy (Kötter et al., 2021). B types were also the most likely to be frequently absent from work/school (due to sickness) (Albisser and Kirchhoff, 2007; Awenius, 2019). In contrast, the most favorable career prospects were found in G-type students, who not only rated their career prospects the highest (Rothland, 2011) but also displayed the highest levels of work-related vigor, dedication and absorption along with pedagogical, psychosocial and intercultural competence (Nolle, 2013; Meier, 2015; Meiseneder, 2015; Genkova and Schreiber, 2021). Further, G-type teacher education students displayed the highest match between their own individual interests and the requirements of teacher education (Kaub et al., 2014), and they expected the highest levels of success in their future career development (Rothland, 2011; Cramer, 2012; Rumpler, 2013). In contrast to their counterparts assigned to the less desirable patterns, G types manifested the lowest tendency toward work avoidance (Cramer, 2012). Finally, along with S types, G-type teacher education students were aware of their resilience to work-related demands and suitability for a teaching career (Schröder and Kieschke, 2006).

### 4.10. Subjective stress and coping strategies

In accordance with the theoretical underpinnings of the WCEP typology, G and S types reported the lowest levels of subjective stress and psychological burden (Schröder and Kieschke, 2006; Voltmer et al., 2012; Rumpler, 2013; Jäger, 2017; Voltmer et al., 2021b; Afshar et al., 2022). On the other side, students assigned to the risk patterns tended to perceive the highest levels of stress–types A were likely to be the most affected by general psychological stress (Aster-Schenck et al., 2010; Rumpler, 2013; Afshar et al., 2022) and stress induced by social commitments, scheduling of the daily routine, work-life balance and partnership problems (Schröder and Kieschke, 2006). During the COVID-19 pandemic, A types were also the most afraid of contagion and felt the largest negative impact of the pandemic on several areas of their life (Voltmer et al., 2021b). B types, on the other side, tended to perceive the highest levels of stress in relation to financial and living conditions or interpersonal conflicts (Schröder and Kieschke, 2006) and regarded their lives as being unpredictable, uncontrollable and overloaded (Voltmer et al., 2021b).

To deal with stress, S–and especially G-type students applied the most productive and health-promoting strategies, such as thinking positively, eating a balanced diet, exercising regularly, getting enough sleep and seeking social support from friends and family. On the other hand, students assigned to the risk patterns tended to use dysfunctional coping strategies, such as denial, self-blame, smoking or drinking alcohol. Ironically, they were also the least interested in information about health-promoting strategies (Buss, 2002; Albisser and Kirchhoff, 2007; Wolf et al., 2007; Jäger, 2017; Voltmer et al., 2021a,b; Afshar et al., 2022).

### 4.11. Physical and mental health

The evidence on the physical and mental health in students assigned to distinct patterns is not surprising in view of the previous paragraph. Applying the theoretical framework of WCEP, it was consistently shown that A- and especially B-type students admitted to having overall bad physical health compared to G and especially S types, who tended to have better physical health and lower levels of physical complaints (e.g., Hamdan, 2012; Voltmer et al., 2012). Likewise, in terms of mental health, G and S types enjoyed the best status. The worst results, on the other hand, were displayed by A- and especially B-type students (Voltmer et al., 2012; Awenius, 2019; Voltmer et al., 2021b). Specifically, G types felt the least socially disconnected and alienated from the rest of the word (Awenius, 2019), and along with S types displayed lower levels of anxiety, depression, exhaustion and cynicism than their counterparts assigned to the risk patterns (Albisser and Kirchhoff, 2007; Obst and Kötter, 2020; Voltmer et al., 2021a,b).

## 5. Discussion

This review, based on 69 records published between 2002 and 2022, aimed to offer a comprehensive overview of the findings on WCEP and their correlates in university students. Specifically, the main categories of correlates identified in student focused WCEP research, which were covered in the present review, were gender, study field, study phase, background characteristics, learning and academic achievement, personality factors, study and career choice motivation, commitment to students’ career choice, suitability for the future profession, subjective stress and coping strategies and physical and mental health.

First, this review revealed that the female students were more likely to be assigned to risk patterns, especially the excessively ambitious risk pattern A than the male students, who in contrast, were more likely to be assigned to the unambitious pattern S. These findings are in accordance with evidence on the WCEP distribution in professionals, such as teachers and physicians (e.g., Schaarschmidt, 2005; Voltmer et al., 2010b), as well as general findings on increased psychological vulnerability in female university students (Auerbach et al., 2018; Sheldon et al., 2021). Our findings also support the notion of a relative stability of the patterns or rather a spontaneous tendency to transition toward less desirable patterns in the long-term suggested by Kieschke and Schaarschmidt (2008), as that the WCEP distribution tended to either persist or transition toward less desirable patterns during the course of study. This outcome is supported by findings from 3-year longitudinal studies of European undergraduate students that suggest a slight but notable worsening of psychological well-being and mental health across the degree course (Bewick et al., 2010; Macaskill, 2013). Background protective factors against being assigned to the less desirable patterns were mainly social and financial support, along with the socioeconomic status of students parents. These findings corroborate previous evidence on the lack of social support, financial difficulties, growing up in a poor family and lower parental educational attainment to increase the likelihood of mental health problems in university undergraduates (Eisenberg et al., 2007; Assari, 2018; Sheldon et al., 2021).

On the other hand, the findings on differences between students of distinct study fields are inconclusive. Although teacher education students seem to display a lower tendency toward the risk patterns than students of other study fields, unequivocal conclusions cannot be drawn due to the underrepresentation of students of study fields other than medicine and teacher education. In this respect, the only clear finding of the present review was that teacher education students were assigned to the risk patterns more frequently than medical students, which is in line with the findings on less desirable WCEP distribution in teachers compared to physicians (Voltmer et al., 2011a). However, embedding these findings into the existing literature on student well-being and mental health is less straightforward due to the lack of clear evidence on differences among academic disciplines. Although it was repeatedly shown that students in art and humanities tend to mark the highest and students in business, engineering and nursing the lowest end of the continuum of various mental health issues, there are mixed research findings with regard to other study fields (Lipson et al., 2016; Erekson et al., 2022; Allen et al., 2022a). Similarly, the link between WCEP and academic achievement is ambiguous. Although healthy ambitious G types tended to achieve better academically, the differences between students assigned to distinct patterns were insignificant. In this respect, mixed results were also provided by research studies on the link between academic achievement and aspects of coping, well-being and burnout. While some studies have shown a positive link (Antaramian, 2015; Thomas et al., 2017), other have provided inconclusive results (Topham and Moller, 2011; Da Silva et al., 2022), or even evidence that high achieving students are more vulnerable to burnout (Kotzé and Kleynhans, 2013; Atalayin et al., 2015).

Regarding personality traits, study and career choice motivation, commitment to the chosen career, suitability for the profession, coping strategies and physical and mental health, the results of the present review are clear and coherent. Evidence consistently shows that the healthy ambitious pattern G was associated with the most desirable correlates, such as adaptive personality traits (e.g., extraversion, conscientiousness and self-efficacy), higher quality motivation, commitment to the chosen career, suitability for the profession, stress resistance, adaptive coping and better physical and mental health. In contrast, the findings on patterns S and A tended to be less straightforward. Generally, S types lacked professional motivation but tended to be healthier and more resistant to stress while A types, although highly motivated, were more vulnerable to stress and suffered from poor health. The least desirable correlates were related to pattern B, with B-type students clearly showing less adaptive personality traits, a tendency to be unmotivated, uncommitted to their chosen career, unsuitable for the profession, vulnerable to stress, unable to cope in a productive way and suffering from poor mental and physical health. This evidence clearly corresponds to the general literature on student burnout. For instance, students who scored high on burnout dimensions displayed high levels of neuroticism, lower quality motivation, reduced career choice satisfaction, tended to high-risk alcohol drinking and substance use and suffered from mental health problems. In contrast, their psychologically less vulnerable counterparts displayed higher levels of extraversion, openness, optimism, self-efficacy, spirituality, proactive personality traits, resilience, adaptive coping strategies, higher quality motivation or career choice satisfaction (Kovach Clark et al., 2009; Pisarik, 2009; Morgan and De Bruin, 2010; Wachholtz and Rogoff, 2013; Vizoso et al., 2019; Gong et al., 2021; Kong et al., 2021; Allen et al., 2022b).

Finally, it is important to highlight that several findings of this review could be considered particularly alarming. First, both A- and B-type students tended to suffer from various physical and mental issues, which implies that the health impairments related to these risk patterns can develop even before the individuals enter the profession. Second, B-type students clearly lacked the necessary prerequisites for their future profession, such as pedagogical and psychosocial competence in teaching or empathy in medicine. This may be taken as an early indication of the impaired quality of future work-related outcomes found in B-type in-service teachers and health care professionals (Klusmann et al., 2006; Mroczek et al., 2018), as well as in professionals with symptoms of burnout (Halbesleben and Rathert, 2008; Madigan and Kim, 2021).

### 5.1. Limitations

The main limitation of the present review is that the included studies provide unbalanced data since German and teacher education students are overrepresented. Further, the included literature varies in quality. Since the present review aimed to offer a comprehensive overview of the literature that provides evidence on WCEP and their correlates, quality-based exclusion was intentionally not performed to encompass all the relevant references.

### 5.2. Directions for future research

It is important for future research to extend WCEP research beyond Germany and German-speaking countries to increase the generalisability of the findings. Further, it would be useful to gain more data on students of other specializations since previous research on WCEP has focused mostly on students of psychologically demanding occupations (teaching and health care). We also suggest to direct future research toward a more concentrated focus on specific background factors (such as, for instance, study history or type of secondary school) linked to the assignment to (un)favorable patterns. Although so far only marginal interest has been devoted to these factors, this evidence can greatly facilitate the early recognition of vulnerable individuals in university settings. In addition, Manzano-García and Ayala (2017) identified several factors that have been neglected in the burnout literature despite their potential significant role in explaining burnout. Among these factors, for example, personality factors, such as self-esteem or problem-solving skills, may be relevant for future student-focused WCEP research.

### 5.3. Conclusions and implications

This review has provided robust evidence about WCEP and their correlates in university students that is largely in line with the literature on student mental health and burnout. In this respect, we can conclude that female students, teacher education students (compared to medical students) and students who receive insufficient social and financial support are at greater risk of being assigned to risk work-related patterns that indicate vulnerability to burnout and occupational health issues. Moreover, students assigned to these patterns, especially to the resigned (burnout) pattern, are prone to manifest other negative characteristics, such as less adaptive personality traits and coping strategies, vulnerability to stress, lower quality motivation, lack of commitment to the chosen career and suitability for the profession and impaired physical and mental health.

The findings of the present review highlight that particular attention must be devoted to psychologically vulnerable individuals in university settings. Specifically, measures for fostering health-promoting coping and behavioural patterns should be adopted early to correct the risk patterns especially in individuals who aim at pursuing psychologically demanding careers. The present review has strong practical implications for higher education institutions, as it significantly contributes to the understanding of the risk and protective factors of vulnerability to burnout and occupational health issues in university students. The evidence provided could therefore be indicative for the selection and development of prospective professionals already in the higher education phase.

## Author contributions

The author confirms being the sole contributor of this work and has approved it for publication.

## Conflict of interest

The author declares that the research was conducted in the absence of any commercial or financial relationships that could be construed as a potential conflict of interest.

## Publisher’s note

All claims expressed in this article are solely those of the authors and do not necessarily represent those of their affiliated organizations, or those of the publisher, the editors and the reviewers. Any product that may be evaluated in this article, or claim that may be made by its manufacturer, is not guaranteed or endorsed by the publisher.
