# Supplementary material for: Work-related coping behaviour and experience patterns in university students: a review of 20 years of research
Source: Front Psychol. 2023 Apr 18;14:1062749. doi: 10.3389/fpsyg.2023.1062749 (PMC10151672; doi:10.3389/fpsyg.2023.1062749)
Supplement: Supplementary file 1 [file Table_1.docx]

Supplementary Material

# Supplementary Table

*A Summarised Overview of Published Studies on Work-Related Coping Behaviour and Experience Patterns (WCEP) in University Students*

| Reference | Country (Institution) | Data Collection Period | Sample  Characteristics | Pattern Distribution  (%) | | | | Findings on WCEP Correlates | QA |
| --- | --- | --- | --- | --- | --- | --- | --- | --- | --- |
|  |  |  |  | G | S | A | B |  |  |
| Abujatum et al. (2007) | Germany | 2001 - 2006 | teacher education students  *n* = 366, females = 79.5%, mean age = 23.5 years  (pre-intervention data) | 25 | 28 | 22 | 25 | N/A | N/A |
| Afshar et al. (2022) | Germany  (Hannover Medical School) | 2018 - 2019 | first- to sixth-year medical students  *n* = 591, females = 75.8%, mean age = N/A | 20 | 22 | 40 | 18 | phase of study: first-year compared to sixth-year (S < A), gender: males compared to females (A < G), receipt of financial support (B < S), substance use (G/S < A/B), self-perceived stress (S < A), perceived medical school stress (G < S < A < B); marital status, children, other vocational training/study (G = S = A = B) | good |
| Albisser & Kirchhoff (2007) | Switzerland | N/A | final year primary teacher education students  *n* = 105, females = 90.5%, mean age = N/A | 41 | 24 | 13 | 22 | frequency of sickness absence (G/S < B), physical complaints (S < A/B), tendency towards depression (G/S < A/B), satisfaction with studies (B < G/S), commitment to pupils (B < G/S/A), striving for competence (B < G), striving for appreciation by pupils/colleagues/school leaders (S/B < G), task-oriented coping (S/B < G), emotion-oriented coping (G/S < B); commitment to colleagues/school leaders/public, disengagement- and distraction-oriented coping (G = S = A = B) | N/A |

| Reference | Country (Institution) | Data Collection Period | Sample  Characteristics | Pattern Distribution  (%) | | | | Findings on WCEP Correlates | QA |
| --- | --- | --- | --- | --- | --- | --- | --- | --- | --- |
|  |  |  |  | G | S | A | B |  |  |
| Aster-Schenck  et al. (2010) | Germany (University of Würzburg) | 2009 | medical students in the 2^nd^, 5^th^, and 10^th^ term  *n* = 360, females = 58%,  mean age = 23.43 years  (SD = 2.50) | 30 | 34 | 16 | 20 | phase of study: initial phase compared to more advanced phases (S/B < G), subjective academic achievement  (S/B < G), subjective psychological burden (G < A/B), subjective health (B < G); age, high school final grade, gender, nationality, attending a course focused on effective examination preparation, marital status (G = S = A = B) | good |
| Awenius (2019) | Germany and Austria | 2018 | students of various fields  *n* = 533, females = 79.5%,  mean age = 23.68  (SD = 2.79) | 16 | 29 | 44 | 11 | unrelenting standards (the underlying belief that one must strive to meet very high internalized standards of behaviour and performance, usually to avoid criticism) (B < G/S/A), enmeshment (G/S/B < A), pessimism (S < A), punitiveness (A < G/S/B), social isolation (feeling disconnected and alienated from the rest of the world) (G < S/A/B), physical and mental health (B < A < G < S) (an insignificant tendency), absenteeism (S < A < B), presenteeism (S < A < B) (an insignificant tendency); type of higher education institution, field of study, gender, nationality (Germany x Austria), other domains of Young Schema Questionnaire, study satisfaction (G = S = A = B) | mod. |
| Bauer (2019) | Germany (University of Köln) | 2011 - 2012 | teacher education students *n* = 779, females = 81%, mean age = 23.92 years | 23 | 38 | 15 | 24 | gender: males compared to females (A/B < G/S), teacher education specialization: grammar school (Gymnasium) compared to other specializations (G/S < A/B), tendency towards irritation (G/S < A/B), psychological complaints (G/S < A/B), self-efficacy (A/B < G/S), uncertainty tolerance (A/B < G/S), mindful attention and awareness (A/B < G/S); exceeding standard length of study, teacher education is the desired study option (G = S = A = B) | good |

# Supplementary Table

*Continued*

# Supplementary Table

*Continued*

| Reference | Country (Institution) | Data Collection Period | Sample  Characteristics | Pattern Distribution  (%) | | | | Findings on WCEP Correlates | QA |
| --- | --- | --- | --- | --- | --- | --- | --- | --- | --- |
|  |  |  |  | G | S | A | B |  |  |
| Beer & Benieschek  (2012) | Austria (University College for Teacher Education of Christian Churches Vienna/Krems) | 2010 - 2011 | teacher education students  in the 3^rd^ and 5^th^ term *n* = 262, females = 90,8%,  mean age = N/A | 44 | 16 | 27 | 13 | N/A | good |
| Böckelmann et al. (2020) ^a^ | Germany | N/A | students of German origin  *n* = 114, females = 39.5%,  mean age = 21.7 years  (SD = 3.5) | 30 | 25 | 20 | 25 | potentially stressful general conditions (working alongside studying, family responsibilities, long distance travelling)  (G = S = A = B) | mod. |
|  |  |  | international students  *n* = 80, females = 45%, mean age = 24.7 years (SD = 2.4) | 32 | 54 | 10.5 | 3.5 |  |  |
| Boxhofer  (2013, 2014) ^c^ | Austria (Private University of Education, Diocese of Linz) | 2006 - 2007 | third-term teacher education students  *n* = 267, females = 78,7%,  mean age = N/A | 67 | 8 | 16 | 9 | teacher education specialization: special school compared to other specializations (G < S) | poor |
| Buss (2002) | Germany (Goethe University Fraknfurt) | 2001 | teacher education students  *n* = 150, females = N/A, mean age = N/A | 17 | 42 | 13 | 28 | teacher education specialization: primary school/Gymnasium compared to Haupt-/Realschule  (G/S < A/B), interest in the topic of stress (B < G/A); gender, age, phase of study (G = S = A = B) | N/A |

# Supplementary Table

*Continued*

| Reference | Country (Institution) | Data Collection Period | Sample  Characteristics | Pattern Distribution  (%) | | | | Findings on WCEP Correlates | QA |
| --- | --- | --- | --- | --- | --- | --- | --- | --- | --- |
|  |  |  |  | G | S | A | B |  |  |
| Çelebi et al. (2014); Schaefer (2012) ^c^ | Germany (University of Potsdam) | 2008 - 2010 | teacher education students  *n* = 293, females = 82%, mean age = 25.10 years  (SD = 3.72)  (pre-intervention data) | 32.1 | 32.4 | 15.4 | 20.1 | N/A | poor |
| Cramer (2012) | Germany, Baden-Württenberg | 2007 - 2008 | teacher education students,  *n* = 393, females = 85.1%, mean age = 22.85 | 40 | 27 | 19 | 14 | teacher education specialization: primary school/Realschule compared to special school (S < A), gender: males compared to females (A/B < G/S) (an insignificant tendency), socioeconomic index (A/B < G/S) (an insignificant tendency), cultural capital (G < S), social capital (A/B < G), neuroticism (G < A/B), agreeableness (G < S), consciousness (S/B < G), performance-avoidance goal orientation (S < G), work avoidance (G < S), being opposed against teaching style experienced as a pupil (G < B), subjective significance of subject-specific study (S/B < G), expectation of success on the school placement (A/B < G/S), perceived success on the school placement (B < S < A < G); field of study, age (G = S = A = B) | good |
|  | Germany (University of Tübingen) | 2007 - 2008 | dentistry students, *n* = 41, females = 57.1%,  mean age = 22.75 | 49 | 2 | 34 | 15 | N/A |  |

# Supplementary Table

*Continued*

| Reference | Country (Institution) | Data Collection Period | Sample  Characteristics | Pattern Distribution  (%) | | | | Findings on WCEP Correlates | QA |
| --- | --- | --- | --- | --- | --- | --- | --- | --- | --- |
|  |  |  |  | G | S | A | B |  |  |
| Deiglmayr et al. (2018) | Switzerland | 2013 | Grammar school (Gymnasium)-specialized teacher education students *n* = 192, females = 39%, mean age = 24.2 years  (SD = 5.2) | 48 | 31 | 13 | 8 | gender: males compared to females (S/A < G/B), field of study: natural sciences compared to mathematics (A < S), health complaints (G/S < A/B), self-perceived suitability for the teaching profession with regard to typical professional requirements (B < S/A < G), constructivist belief (belief about learning as an active and self-directed process that leads the teacher to design cognitively activating lessons) (B < S/A < G) | mod. |
| Dietrich et al. (2015) | Germany | 2014 | teacher education students *n* = 430, females = 59%, mean age = 23.95 years (SD = 3.30) | N/A | N/A | N/A | N/A | stability (in terms of resilience) (A/B < G/S), the highest is the match between individual scores and pattern S, the lowest is the motivation for choosing a teaching career due to subject-specific interests | N/A |
| Fischer et al. (2018, 2022) ^c^ ^d^ | Germany | N/A | sport students  *n* = 851, females = 33.3%,  mean age = 21.11 years  (thereof 225 physical education-specialized teacher education students,  females = 37.9%) | 42 | 14 | 26 | 18 | study program: physical education compared to sport studies (A/B < S), gender: males compared to females  (A < G) | poor |
| Genkova & Schreiber (2019, 2020, 2021) ^c^ | Germany | N/A | students of various fields  *n* = 202, females = 52.5%,  mean age = N/A | 40 | 27 | 20 | 12 | motivational intercultural competence (desire to become familiar with the new culture, adapt, and master challenges) (A/B < G/S); meta-cognitive, cognitive, and behavioural intercultural competence (G = S = A = B) | poor |

# Supplementary Table

| Reference | Country (Institution) | Data Collection Period | Sample  Characteristics | Pattern Distribution  (%) | | | | Findings on WCEP Correlates | QA |
| --- | --- | --- | --- | --- | --- | --- | --- | --- | --- |
|  |  |  |  | G | S | A | B |  |  |
| Grözinger & Föster (2016) | Germany (University of Flensburg) | 2009 - 2012 | teacher education students *n* = 1370, females = 79.8%, mean age = 22.65 years  (SD = 4.04) | 33 | 21 | 24 | 22 | phase of study: initial compared to more advanced  (G/A/B < S) | good |
| Hamdan (2012) ^a, *^ | Germany (Saarland University) | 2007 | third-term medical students  *n* = 100, females = 62%,  mean age = 22.4 years  (SD = 2.84)  (t1 data) | 69 | 5 | 24 | 2 | anxiety (S < G < B < A), depression (G < S < B < A), exhaustion (S < G < A < B), stomach complaints  (S < G < A < B) (an insignificant tendency), aching limbs  (S < G < A < B), heart complaints (S < G < A < B), pressure caused by complaints (S < G < A < B), perceived social support (A < S < B < G); traumatic experience  (G = S = A = B) | mod. |
|  |  |  | sixth-term medical students  *n* = 78, females = 66.7%, mean age = 25.46 years  (SD = 2.08) | 78 | 7 | 14 | 1 | anxiety (G < S < A < B), depression (B < G < A < S), exhaustion (G < S < B < A), stomach complaints  (S < G < A < B) (an insignificant tendency), aching limbs  (B < S < G < A), heart complaints (B < G < S < A) (an insignificant tendency), pressure caused by complaints  (S < G < B < A), perceived social support (B < A < S < G)  (an insignificant tendency); traumatic experience  (G = S = A = B) |  |

*Continued*

# Supplementary Table

*Continued*

| Reference | Country (Institution) | Data Collection Period | Sample  Characteristics | Pattern Distribution  (%) | | | | Findings on WCEP Correlates | QA |
| --- | --- | --- | --- | --- | --- | --- | --- | --- | --- |
|  |  |  |  | G | S | A | B |  |  |
| Jäger (2017) | Germany  (FOM University of Applied Sciences for Economics and Management) | 2015 | part-time economics students  *n* = 497, females = 43.7%, mean age = 23.6 (SD = 4.08) | 54.4 | 18.8 | 18.5 | 8.3 | current stress level (G/S < A/B), physical and mental symptoms of stress (G/S < A/B), positive thinking  (B < S/A < G), active coping (A/B < S < G), social support  (B < A < S < G), alcohol consumption and smoking  (B > A > S > G), age: younger vs. older (S/A/B < G), job status: full-time job vs. non-full-time job (G < S/A/B), number of employees in respondents’ employing company: lower number vs. higher number (G < S/A/B), study motivation: enhancement of professional skills, interest in technical content, recommendation by the employer  (S/A/B < G), employer involved in study-related cost payment (S/A/B < G); support in faith coping style, motivated through interest in personal further education, motivated through social influence, motivated through desire for professional advancement, motivated through other reasons, gender, relationship status, number of hours devoted to studying/working/family and household responsibilities, income, preventive health behaviour  (G = S = A = B) | mod. |
| Kada (2014) | Austria  (Carinthia University of Applied Sciences) | 2011 | part-time first-term nursing students  *n* = 66, females = 82.8%, mean age = 29.1 (SD = 6.5)  (t1 data) | 47 | 15 | 18 | 20 | N/A | poor |

| Reference | Country (Institution) | Data Collection Period | Sample  Characteristics | Pattern Distribution  (%) | | | | Findings on WCEP Correlates | QA |
| --- | --- | --- | --- | --- | --- | --- | --- | --- | --- |
|  |  |  |  | G | S | A | B |  |  |
| Kaub et al. (2014) | Germany (Saarland University) | 2009 - 2011 | first-term teacher education students  *n* = 500, females = 63.2%, mean age = 20.33 years (SD = 2.56) | N/A | N/A | N/A | N/A | congruence of interests (match between individual interests and requirements of a particular study program) (S/B < G) | good |
| Kötter et al. (2021) | Germany  (University of Lübeck) | 2011 - 2017 | medical students  *n* = 565, females = 72%, mean age = 22.9 (SD = 3.09) (t1 data) | N/A | N/A | N/A | N/A | pattern A linked to lower levels of empathy at t1; pattern B at t0 predicted lower levels of empathy at t2 in the longitudinal sample | good |
| Künsting et al. (2012) ^d^ | Germany (University of Kassel) | N/A | first-term teacher education students, *n* = 538,  females = 71.8%,  mean age = 21.15 years  (SD = 3.35) | 30 | 19 | 19 | 32 | gender: males compared to females (A/B < G/S), intrinsic career choice motivation (S/B < G/A), learning goal orientation  (S/B < G/A), use of learning strategies  (S/B < G/A), study satisfaction (B < G/S < A), conscientiousness (S/B < G/A), neuroticism (G/S < A/B), average grades in pedagogical courses (S < A); teacher education specialization, extrinsic career choice motivation, average grades in the main subjects, the number of exams passed by the end of the fourth term  (G = S = A = B) | good |

# Supplementary Table *Continued*

# Supplementary Table

| Reference | Country (Institution) | Data Collection Period | Sample  Characteristics | Pattern Distribution  (%) | | | | Findings on WCEP Correlates | QA |
| --- | --- | --- | --- | --- | --- | --- | --- | --- | --- |
|  |  |  |  | G | S | A | B |  |  |
| Lüftenegger et al. (2019); Rumpler (2013); Würfel (2013) ^c^ | Austria (University of Vienna; University College for Teacher Education in Vienna) | 2012 - 2013 | teacher education students *n* = 182, females = 79.1%, mean age = 24.28 years (SD = 5.39) | 28.6 | 29.7 | 22.5 | 19.2 | neuroticism (G/S < A < B), extraversion (A/B < G), conscientiousness (S/B < G/A), time point of the decision of becoming a teacher: the life-long anchored decision of pursuing a teaching career compared to the decision of pursuing a teaching career at the start of teacher education  (B < A), decision of pursuing a teaching career at the start of teacher education compared with the decision of pursuing a teaching career at a later point after starting teacher education (G/S/A < B), study satisfaction (B < G), self-rated future career success (B < G) (an insignificant tendency), subjective certainty of choosing teacher education (B < S/A), perceived burden in everyday life (G/S < A/B), educational level of father: elementary school compared with higher educational levels (G/S/A < B), number of hours/week devoted to studying  (S/B < A), level of financial support (A/B < S) (an insignificant tendency); openness, agreeableness, self-rated career prospects, plans after graduation, educational level of mother, social support, teacher education specialization, type of higher education institution (university x university college)  (G = S = A = B) | mod. |

*Continued*

# Supplementary Table

*Continued*

| Reference | Country (Institution) | Data Collection Period | Sample  Characteristics | Pattern Distribution  (%) | | | | Findings on WCEP Correlates | QA |
| --- | --- | --- | --- | --- | --- | --- | --- | --- | --- |
|  |  |  |  | G | S | A | B |  |  |
| Manderfeld & Siller (2019) | Germany | 2017 | teacher education students  *n* = 146, females = 76%, mean age = 22.25  (SD = 2.38) | 45.9 | 35.6 | 13.7 | 4.8 | specialization of teacher education: primary vs. secondary school (G = S = A = B) | poor |
| Martin (2012) | Germany (University of Paderborn) | 2012 | physical education-specialized teacher education students  *n* = 125, females = 36%,  mean age = 22.2 years  (SD = 2.4) | 31 | 40 | 11 | 18 | gender: males compared to females (G/A/B < S); specialization of teacher education (G = S = A = B) | mod. |
| Mašková et al. (2022) ^a^ | Germany (University of Passau) | 2019 | first-term teacher education students  *n* = 216, females = 80%,  mean age = 19.55 years  (SD = 2.36) | 37 | 31 | 13 | 19 | gender: males compared to females (G < S), job status: having a personally important job compared to non-personally important job (B < G), study history: being enrolled on the first and only study program compared with non-first and only study program (B < G), intrinsic motivation (A < G), identified regulation  (S/B < G); teacher education specialization, introjected regulation, external regulation, career choice motivation (G =S = A = B) | good |
|  | Czech Republic (University of South Bohemia) | 2019 | first-term teacher education students  *n* = 265, females = 61%,  mean age = 20.22 years  (SD = 2.23) | 17 | 31 | 19 | 33 | job status: having a personally important job compared to a non-personally important job (B < G), study history: being enrolled on the first and only study program compared with non-first and only study program (B < G), intrinsic motivation (A/B < G), identified regulation (S/B < G); introjected regulation, external regulation, career choice motivation (G = S = A = B) |  |

# Supplementary Table

| Reference | Country (Institution) | Data Collection Period | Sample  Characteristics | Pattern Distribution  (%) | | | | Findings on WCEP Correlates | QA |
| --- | --- | --- | --- | --- | --- | --- | --- | --- | --- |
|  |  |  |  | G | S | A | B |  |  |
| Meier (2015) | Germany, North Rhine-Westphalia | 2010 - 2011 | teacher education students  *n* = 681, females = 56.4%, mean age = 28.0 years  (SD = 4.4)  (t1 data collected shortly after completing the first phase of teacher training) | 39 | 37 | 11 | 13 | gender: males compared to females (A < G/S/B), self-rated pedagogical competencies (B < G); age, teacher education specialization (G = S = A = B) | good |
| Meiseneder (2015) | Austria (University of Vienna) | 2014 | psychology students  *n* = 106, females = 80.2%, mean age = 26.1 (SD = 4.2) | 34 | 29.2 | 17.9 | 18.9 | work engagement (vigour, dedication, and absorption)  (S < B < A < G), self-efficacy (B < A < S < G) | mod. |
|  | Austria  (University College for Teacher Education of Christian Churches in Krems) | 2015 | teacher education students  *n* = 107, females = 87.9%,  mean age = 23.6 (SD = 4.6) | 38.3 | 28 | 22.4 | 11.2 |  |  |
| Nolle (2013) | Germany (University of Kassel) | 2008 | first-term teacher education students  *n* = 564, females = 73.4%, mean age = 21.2 years  (SD = 3.42) | 26.7 | 38.5 | 16.1 | 18.6 | psychosocial competence and learning orientation (openness to situations that enable personal growth)  (S/A/B < G) (an insignificant tendency) | good |

*Continued*

# Supplementary Table

*Continued*

| Reference | Country (Institution) | Data Collection Period | Sample  Characteristics | Pattern Distribution  (%) | | | | Findings on WCEP Correlates | QA |
| --- | --- | --- | --- | --- | --- | --- | --- | --- | --- |
|  |  |  |  | G | S | A | B |  |  |
| Nowik & Franke (2009) ^a^ | Germany (Magdeburg-Stendal University of Applied Sciences) | N/A | rehabilitation psychology students *n* = 67, females = 85.1%, mean age = 23 years  (SD = 2.8) | 43 | | 57 | | N/A | poor |
|  |  |  | business administration students *n* = 40, females = 70%, mean age = 22 years (SD = 2.58) | 62 | | 38 | | N/A |  |
| Nusseck & Spahn (2013) | Germany (Freiburg University of Music) | N/A | music students  *n* = 70, females = 61%, mean age = 25.1 years (SD = 1.9) | 32 | 31 | 14 | 23 | study program: music teacher education compared with musical art (A < S) (an insignificant tendency) | poor |
| Obst et al. (2017) | Germany  (University of Lübeck) | 2015 | first- to third-year medical students, *n* = 336,  females = 69%,  mean age = 22.57  (SD = 2.93) | 39.3 | 26.2 | 21.4 | 12.8 | identification with the medical study programme  (S/B < G/A), health status (A < G), anxiety (G < A), depression (G < A) | good |
| Obst & Kötter (2020) | Germany (University of Lübeck) | 2015 | natural science students *n* = 378, females = 62.5%, mean age = 21.46 years (SD = 2.20) | 18 | 26.5 | 25.5 | 30 | identification with the study programme (B < G/A), health status (B < G/S), anxiety (G/S < A/B), depression  (G/S < B) | good |

# Supplementary Table

*Continued*

| Reference | Country (Institution) | Data Collection Period | Sample  Characteristics | Pattern Distribution  (%) | | | | Findings on WCEP Correlates | QA |
| --- | --- | --- | --- | --- | --- | --- | --- | --- | --- |
|  |  |  |  | G | S | A | B |  |  |
| Poterpin et al. (2020) | Austria (University College for Teacher Education in Vienna) | 2018 | first-term primary teacher education students  *n* = 256, females = N/A, mean age = N/A | 34 | 29 | 16 | 21 | N/A | N/A |
| Reichl et al.  (2014) ^a^ | Germany | N/A | teacher education students *n* = 559, females = 62.6%, mean age = 21.16 years  (SD = 3.16) | 26 | 28 | 23 | 23 | neuroticism (G < A/B), extraversion (S/A/B < G), agreeableness (G < S), conscientiousness (S/B < G), motivation in choosing teacher education due to the subject-specific interest (S < G), motivation in choosing teacher education due to the low difficulty of the studies  (G < A/B); openness, motivation in choosing teacher education due to educational interests, ability beliefs, utility, and social influences (G = S = A = B) | good |
|  |  |  | psychology students *n* = 150, females = 72%, mean age = 21.89 years (SD = 4.10) | 18.7 | 26.7 | 16 | 38.7 | neuroticism (G < A/B), extraversion (S/A/B < G), openness  (A < G), agreeableness (A/B < G < S), conscientiousness  (S/B < G) |  |
| Ritter (2017) | Germany (University of Lübeck) | 2012 | first-year medical students  *n* = 102, females = 69%,  age = 21.4 years (SD = 2.90)  (t1 data) | 33 | 24 | 28 | 15 | N/A | good |

# Supplementary Table

*Continued*

| Reference | Country (Institution) | Data Collection Period | Sample  Characteristics | Pattern Distribution  (%) | | | | Findings on WCEP Correlates | QA |
| --- | --- | --- | --- | --- | --- | --- | --- | --- | --- |
|  |  |  |  | G | S | A | B |  |  |
| Roloff Henoch et al. (2015) ^a, d^ | Germany, Baden-Württemberg | 2006 | teacher education students  *n* = 264, females = 80.6%,  mean age = 23.45 years  (SD = 0.60)  (t1 data) | 37 | 21 | 12 | 30 | N/A | good |
|  |  |  | students of other study fields  *n* = 1594, females = 57.2%, mean age= 23.45 years (SD = 0.55)  (t1 data) | 43 | 17 | 18 | 22 | N/A |  |
| Römer et al. (2012, 2013) ^a, c^ | Germany (Goethe University Frankfurt) | 2010 - 2011 | first- and second-term teacher education students  *n* = 293, females = 73.5%, mean age = N/A | 29 | 34.5 | 15 | 21.5 | N/A | mod. |
|  |  |  | first- and second-term law students  *n* = 348, females = 68.1%,  mean age = N/A | 43.5 | 19 | 21.5 | 16 | N/A |  |
|  |  |  | teacher education students (all terms), *n* = 763, females = 77%, mean age = 24.4 years (SD = 3.14) | 29.5 | 29 | 19.5 | 22 | gender, phase of study (term in which students were enrolled), teacher education specialization (G = S = A = B) |  |
|  |  |  | law students (all terms), *n* = 651, females = 67%, mean age = 23.6 years (SD = 2.89) | 36.5 | 17 | 25 | 21.5 | gender: males compared to females (G/A < S), phase of study: initial compared to more advanced (A/B < G) |  |

| Reference | Country (Institution) | Data Collection Period | Sample  Characteristics | Pattern Distribution  (%) | | | | Findings on WCEP Correlates | QA |
| --- | --- | --- | --- | --- | --- | --- | --- | --- | --- |
|  |  |  |  | G | S | A | B |  |  |
| Römer et al. (2017) | Germany (University of Erfurt) | N/A | teacher education students in first and second year of a master’s degree course *n* = 131, females = 85%, mean age = 24.08 years (SD = 2.92) | 39 | 23 | 23 | 15 | neuroticism (G/S < A/B), extraversion (S/A/B < G), consciousness (S/B < A/G), increase of pedagogical knowledge during the final phase of studies (S < A);  openness, agreeableness, level of pedagogical knowledge  (G = S = A = B) | mod. |
| Rothland (2011, 2012, 2013); Rothland et al. (2014) ^c^ | Germany (University of Bochum, University of Erfurt, University of Münster, University of Osnabrück, University of Paderborn) | 2010 | teacher education students *n* = 1142, females = N/A, mean age = 23.66 years (SD = 2.86) | 35 | 33 | 16.5 | 15.5 | study satisfaction (B < S/A < G), self-rated career prospects  (B < S/A < G), self-rated future career success  (B < S/A < G), gender: males compared to females (A < S), phase of study: 5th - 8th term compared to > 12th term  (B < G), teacher education specialization: Sekundarstufe II compared to other study specializations (S < G), subjective certainty of choosing teacher education (A/B < G), time point of the decision of becoming a teacher: life-long anchored decision compared to a decision made at the beginning of teacher education (S/B < G), teacher education as a study of second option (men only)  (G/S/A < B), plans for after graduation: intention to start a teaching career as fast as possible compared to the lack of intention in pursuing a teaching career (A/B < G); motivation in choosing teaching career: the perceived teaching abilities  (B < S < G), the intrinsic value  (B < S < G), as a fallback career (G < B), job security  (S < G/A/B), time for family (G < S/A/B), to shape the future of children/adolescents (B < S < A < G), to enhance social equity (S < G < A),  (*continues*) | good |

# Supplementary Table

*Continued*

# Supplementary Table

*Continued*

| Reference | Country (Institution) | Data Collection Period | Sample  Characteristics | Pattern Distribution  (%) | | | | Findings on WCEP Correlates | QA |
| --- | --- | --- | --- | --- | --- | --- | --- | --- | --- |
|  |  |  |  | G | S | A | B |  |  |
| Rothland (2011, 2012, 2013); Rothland et al. (2014) ^c^ | Germany (University of Bochum, University of Erfurt, University of Münster, University of Osnabrück, University of Paderborn) | 2010 | teacher education students *n* = 1142, females = N/A, mean age = 23.66 years (SD = 2.86) | 35 | 33 | 16.5 | 15.5 | (*continued*)  to make a social contribution (B < S < G < A), to work with children/adolescents (B < G), previous teaching and learning experiences (B < G), the belief that teaching career requires high levels of expertise (S < B < G < A), is a highly demanding career (S < A), is related to high social status  (B < S < G); experienced social dissuasion from choosing a teaching career (S < A), satisfaction with the choice of a teaching career (B < S < G); motivation in choosing a teaching career due to social influences, the belief that a teaching career is related to a good salary (G = S = A = B) | good |
| Schaarschmidt (2005, 2012) | Germany | 2000 - 2005 | teacher education students *n* = 622, females = 79.1%, mean age = 22.65 years  (SD = 2.8) | 29 | 31 | 15 | 25 | certainty about teaching being the right career (B < G) | N/A |
| Scholz et al. (2015) ^b^ | Germany | 2012 - 2013 | medical students in the 1^st^, 2^nd^, 3^rd^, and 4^th^ terms  *n* = 530,  females = 55.4%,  mean age = 22.1 years  (SD = 3.42) | 38 | 25 | 21 | 16 | phase of study: initial phase compared to more advanced phases (A/B < G/S) | good |

# Supplementary Table

*Continued*

| Reference | Country (Institution) | Data Collection Period | Sample  Characteristics | Pattern Distribution  (%) | | | | Findings on WCEP Correlates | QA |
| --- | --- | --- | --- | --- | --- | --- | --- | --- | --- |
|  |  |  |  | G | S | A | B |  |  |
| Schröder & Kieschke (2006) ^b^ | Germany (University of Münster, University of Potsdam) | 2001 - 2004 | teacher education students  *n* = 774, females = 80%,  mean age = N/A | 23 | 30 | 21 | 26 | phase of study: initial phase compared to the advanced phase (A/B < G/S), university: Potsdam compared to Münster (G < A), perceived burden in everyday life  (G/S < A/B), satisfaction with studies (A/B < G/S), satisfaction with academic achievement (A/B < G/S), dealing with study and methodical-didactical requirements (A/B < G/S), perceived career suitability (A/B < G/S), perceived work-related resilience (A/B < G/S) | poor |
| Störländer et al. (2020) | Germany (Bielefeld University) | 2018 | teacher education students  *n* = 15, females = N/A, mean age = N/A | 46.7 | 20 | 6.7 | 26.7 | N/A | N/A |
| Voltmer et al. (2007); Voltmer, Kieschke & Spahn (2008); Voltmer, Kieschke, Schwappach, et al. (2008) ^a, c^ | Germany (University of Freiburg, University of Lübeck) | N/A | first-year medical students  *n* = 424, females = 62%, mean age = 20.8 (SD = 2.4) | 34.4 | 24.8 | 22.9 | 17.9 | social support (A/B < G/S), self-awareness (S < G < A); university: Freiburg compared to Lübeck (A < S)  gender (G = S = A = B) | good |
|  |  |  | fifth-year medical students  *n* = 335, females = 66.6%, mean age = 24.6 (SD = 2.5) | 25.7 | 41.5 | 9.6 | 23.3 | gender: males compared to females (S < G), social support  (B < G/S/A), self-awareness (S < A) |  |

# Supplementary Table

*Continued*

| Reference | Country (Institution) | Data Collection Period | Sample  Characteristics | Pattern Distribution  (%) | | | | Findings on WCEP Correlates | QA |
| --- | --- | --- | --- | --- | --- | --- | --- | --- | --- |
|  |  |  |  | G | S | A | B |  |  |
| Voltmer et al.  (2010, 2012) ^a, c^ | Germany (University of Lübeck) | 2006 | first-term medical students  *n* = 112, females = 70.5%,  mean age = 20.9 years (SD = 3.2) | 47 | 29 | 17 | 7 | phase of study: 1st year compared to 2nd year (B < G), 2nd year compared to 5th year (G/A < S), gender: males compared to females (A < G) (an insignificant tendency), perceived medical school stress (G/S < A/B), physical health (B < S), mental health (B < G/S), anxiety (G/S < B), depression (G/S < B), self-rated performance (S/B < G); efficacy/efficiency, grades (G = S = A = B) | good |
|  |  | 2008 | fourth-term medical students  *n* = 160, females = 67.7%, mean age = 22.8 (SD = 3.3) | 37 | 27 | 16 | 20 |  |  |
|  |  | 2011 | fifth-year medical students  *n* = 153, females = 71.3%,  mean age = 25.6 (SD = 3.1) | 17.6 | 59.5 | 3.9 | 19 |  |  |
| Voltmer et al. (2011) | Germany (Friedensau Adventist University) | 2007 | theology students  *n* = 44, females = 6.8%,  mean age = 25.8 (SD = 4.5) | 31.7 | 36.6 | 17.1 | 14.6 | mental health (A/B < G/S), daily spiritual experiences  (A/B < G); physical health, other religiosity/spirituality indicators (G = S = A = B) | good |
| Voltmer et al. (2019) | Germany (University of Lübeck) | 2011 - 2016 | first- to third-year STEM students  *n* = 130, females = 56.7%, mean age = N/A  (t0 longitudinal sample data) | 46 | 23 | 21 | 10 | gender: males compared to females (B < G) | good |
| Voltmer, Köslich-Strumann, Voltmer et al. (2021); Voltmer et al.  (2019) ^c^ | Germany (University of Lübeck) | 2011 - 2019 | first- to sixth year medical students,  *n* = 377, females = 70%, mean age = 21.0 (SD = 3.1)  (t0 longitudinal sample data) | 56 | 13 | 22 | 9 | gender: males compared to females (A/B < G), exhaustion  (S/G < A/B), cynicism (G < S < A < B), efficacy  (B < S < A < G), functional coping (A/B < G/S), dysfunctional coping (G/S < B) | good |

# Supplementary Table

*Continued*

| Reference | Country (Institution) | Data Collection Period | Sample  Characteristics | Pattern Distribution  (%) | | | | Findings on WCEP Correlates | QA |
| --- | --- | --- | --- | --- | --- | --- | --- | --- | --- |
|  |  |  |  | G | S | A | B |  |  |
| Voltmer, Köslich-Strumann, Walther et al. (2021) ^a^ | Germany (University of Lübeck) | 2019 | STEM, medical, and health science students  *n* = 890, females = 78.7%, mean age = 24.1 (SD = 3.3)  (longitudinal sample data) | 36 | 26 | 20 | 19 | year of survey: 2019 (pre-pandemic) compared to 2020 (pandemic) (S < G) | good |
|  |  | 2020 | STEM, medical, and health science students  *n* = 890, females = 78.7%, mean age = 24.1 (SD = 3.3)  (longitudinal sample data) | 31 | 31 | 18 | 20 |  |  |
|  |  | 2020 | STEM, medical, and health science students  *n* = 1709, females = 75.2%, mean age = 23.8 (SD = 3.5)  (full sample data) | N/A | N/A | N/A | N/A | fear of contagion with the coronavirus (S < G < B < A); perceived impact of the COVID-19 pandemic on the studies  (S < A/B), perceived impact of the COVID-19 pandemic on economic existence (G/S < A/B), perceived impact of the COVID-19 pandemic on intangible existence (emotions, meaning of life) (G/S < A/B), wearing a mask (S < G/A), washing hands more often (S < B < G/A), taking more care about cleanliness  (S < B < G < A), using disinfectants (S/B < G < A), eating a balanced diet  (B < S/A < G), exercising regularly (A/B < S < G), taking herbal supplements (S/G < A/B), sleeping sufficiently  (A/B < G/S), contacting friends and family  (B < A < S < G), perceived stress (G < S < A < B), somatic symptoms (G/S < A/B), depression (G < S < A < B), anxiety (G/S < A/B) |  |

# Supplementary Table

| Reference | Country (Institution) | Data Collection Period | Sample  Characteristics | Pattern Distribution  (%) | | | | Findings on WCEP Correlates | QA |
| --- | --- | --- | --- | --- | --- | --- | --- | --- | --- |
|  |  |  |  | G | S | A | B |  |  |
| Wild et al. (2014) | Germany | 2012 - 2013 | medical and psychology students from 5^th^ to 8^th^ term  *n* = 42, females = 88%, mean age = 24.44 (SD = 3.14) | 61 | | 39 | | N/A | good |
| Wolf et al. (2007) | Germany (University of Magdeburg) | 2006 | students of various fields *n* = 36, females = 42%,  mean age = N/A | 34.3 | 34.3 | 20 | 11.4 | health status (A < G), coping through friends, family, music, sleeping sufficiently, and eating healthy  (S/A/B < G), coping through alcohol consumption and smoking (G/S/B < A), an opinion that one has to be fit for the labour market (S/A/B < G), being informed about health-promoting strategies (S/A/B < G), interest in information on health-promoting strategies (A < G/S/B) | poor |
| Zehenter & Boxhofer (2018) | Austria (Private University of Education, Diocese of Linz) | 2016 | teacher education students *n* = 224, females = 86.2%, mean age = N/A | 58 | 24 | 12 | 6 | N/A | mod. |

*Continued*

*Note.* This overview involves studies published before September 2022 that employ the AVEM inventory in higher education students and present either the pattern distribution in the sample and/or findings on correlates related to distinct patterns. The studies that use the AVEM inventory in students but do not provide data on the pattern assignment (providing instead only data related to the AVEM scales) were not included. The list of the studies is a result of systematic search, but it may not be exhaustive. Totals of pattern distribution may deviate from 100% due to rounding imprecisions.

Institution refers to university/college university where the participants were enrolled (this applies only if the information was provided); N/A = the respective data is unavailable; Gymnasium refers to grammar school covering both the lower and upper levels (5th – 12th class); Hauptschule and Realschule refer to a school covering lower secondary level; Sekundarstufe II refers to upper secondary education. STEM = science, technology, engineering, and mathematics; QA = Quality assessment outcome: good = good quality; mod. = moderate quality; poor = poor quality.

^a^ The study involves multiple distinct samples, which were included in the present overview. ^b^ The study involves multiple samples, which were pooled for the purposes of the present overview. ^c^ These studies rely on the same dataset. ^d^ In contrast to other studies included in this overview, which assign participants to distinct work-related patterns according to the algorithm provided by the authors of the AVEM inventory, this study employs a latent profile analysis to assign the participant to the patterns.^*^ This study relies on an 11-item short form of AVEM that is intended only as a self-test instrument.

# References

Abujatum, M., Arold, H., Knispel, K., Rudolf, S. & Schaarschmidt, U. (2007). Intervention durch Training und Beratung. In U. Schaarschmidt & U. Kieschke (Eds.),
*Gerüstet für den Schulalltag: Psychologische Unterstützungsangebote für Lehrerinnen und Lehrer* (pp. 157-187). Beltz.

Afshar, K., Wiese, B., Stiel, S., Schneider, N., & Engel, B. (2022). Perceived stress and study-related behavior and experience patterns of medical students: A cross-sectional study. *BMC Medical Education*, *22*(1), 122. https://doi.org/10.1186/s12909-022-03182-4

Albisser, S., & Kirchhoff, E. (2007). Salute! Zur Berufsgesundheitlichen Kompetenzentwicklung Studierender. *Journal für Lehrerinnen- und Lehrerbildung*, *4*, 32-39.

Aster-Schenck, I., Schuler, M., Fischer, M. R., & Neuderth, S. (2010). Psychosoziale Ressourcen und Risikomuster für Burnout bei Medizinstudenten: Querschnittstudie und Bedürfnisanalyse Präventiver Curricularer Angebote. *GMS Zeitschrift für Medizinische Ausbildung*, *27*(4), Doc61. <https://doi.org/10.3205/zma000698>

Awenius, L.-F. O. (2019). *Gesundheitsrelevante Arbeitsmuster als Bewältigungsversuch von frühen maladaptiven Schemata bei österreichischen und deutschen Studierenden in der EmAd-Phase*.

[Master’s Thesis, University of Klagenfurt]. https://netlibrary.aau.at/obvuklhs/content/titleinfo/5336559/full.pdf

Bauer, J. F. (2019). *Personale Gesundheitsressourcen in Studium und Arbeitsleben: Transaktionales Rahmenmodell und Andwendung auf das Lehramt*. Springer.

Beer, G., & Benieschek, I. (2012). Zum Zusammenhang von Persönlichkeitsmerkmalen und Verhaltens- und Erlebensmustern bei Studierenden an Pädagogischen Hochschulen. In I. Benieschek, A. Forstner-Ebhart, H. Schaupp, & H. Schwetz (Eds.), *Empirische Forschung zu schulischen Handlungsfeldern. Erbegnisse der ARGE Bildungsforschung an Pädagogischen*

*Hochschulen in Österreich* (Vol. 2, pp. 153-181). LIT Verlag.

Böckelmann, I., Darius, S., Zavgorodnii, I., & Thielmann, B. (2020). Resource-based strategies for health promotion of students with different general conditions and different origins. *Inter Collegas*, *8*(3), 132-143. https://doi.org/10.35339/ic.8.3.132-143

Boxhofer, E. (2013). *Persönlichkeit und Belastungserleben von Lehramtsstudierenden. Eine Studie im Kontext der schulpraktischen Ausbildung.* [Doctoral Dissertation, University of Klagenfurt]. https://netlibrary.aau.at/obvuklhs/content/titleinfo/2416170

Boxhofer, E. (2014). SPAVEM – Schulpraxisbezogenes Verhaltens- und Erlebensmuster im Kontext der Änderungen von Persönlichkeitsmerkmalen. In I. Benieschek, A. Forstner-Ebhart, H. Schaupp, & H. Schwetz (Eds.), *Empirische Forschung zu schulischen Handlungsfeldern. Erbegnisse der ARGE Bildungsforschung an Pädagogischen Hochschulen in Österreich* (Vol. 4, pp. 21-36). Facultas.

Buss, C. (2002). Persönlichkeitsmuster und Stressbewältigung – Ein Vergleich zwischen LehrerInnen und LehramtsstudentInnen der Johann Wolfgang Goethe Universität.
*L-news*, *17*, 5-14.

Çelebi, C., Krahé, B. & Spörer, N. (2014). Gestärkt in den Lehrerberuf: Eine Förderung berufsbezogener Kompetenzen von Lehramtsstudierenden. *Zeitschrift für Pädagogische Psychologie, 28,* 115–126. https://doi.org/[10.1024/1010-0652/a000128](https://doi.org/10.1024/1010-0652/a000128)

Cramer, C. (2012). Entwicklung von Professionalität in der Lehrerbildung. Empirische Befunde zu Eingangsbedingungen, Prozessmerkmalen und Ausbildungserfahrungen Lehramtsstudierender. Klinkhardt.

Deiglmayr, A., Grabner, R. H., Nussbaumer, D., & Saalbach, H. (2018). Gesund und kompetent: Beanspruchungserleben, gesundheitliche Beschwerden und Berufseignung–Eine Studie mit Schweizer Lehramtsstudierenden. *Beiträge zur*

*Lehrerinnen- und Lehrerbildung*, *36*, 262-281. https://bzlonline.ch/download/279/BzL_182_262-281Deiglmayr.pdf

Dietrich, S., Schoppe, S., Preß, L. & Latzko, B. (2015, September 14–16). *Macht Schonung glücklich? Zusammenhänge zwischen Studienwahlmotivation bzw. Berufsbezogenen Personeneigenschaften und arbeitsbezogenen Verhaltens- und Erlebensmustern sowie Zufriedenheit* [Poster Presentation]. 15. Fachgruppentagung Pädagogische Psychologie, Kassel, Germany

Fischer, B., Bisterfeld, M., & Staab, O. (2018). Individual’s patterns of commitment, resilience and subjective well-being of prospective physical education teachers.

*Australian Journal of Teacher Education*, *43*(2), 39-55. <http://dx.doi.org/10.14221/ajte.2018v43n2.3>

Fischer, B., Poweleit, A. & Konowalczyk, S. (2022). Adaptive Selbstregulation von Sportstudierenden: Unterscheiden sich angehende Sportlehrkräfte von Sportstudierenden mit einem anderen Berufsziel? *German Journal of Exercise and Sport Research.*

https://doi.org/10.1007/s12662-022-00830-1

Genkova, P., & Schreiber, H. (2019). Impact of stays abroad on intercultural competence of students. In B. C. Venegas, C. Debray, & J. Vakkayil (Eds.) *IACCM-IÉSEG 2019 Conference Proceedings* (pp. 80-98). IACCM & IÉSEG.

Genkova, P., & Schreiber, H. (2020). Die Auswirkung eines Auslandsaufenthaltes auf die interkulturelle Kompetenz von Studierenden. In GfA, Dortmund (Ed.) *Frühjahrskongress 2020, Digitaler Wandel, digitale Arbeit, digitaler Mensch* (C.2.5). GfA Press.

Genkova, P., & Schreiber, H. (2021). Stays abroad and intercultural competence of students. *European Journal of Cross-Cultural Competence and Management*, *5*(3), 271-286.

https://doi.org/271-286. 10.1504/EJCCM.2021.116888

Grözinger, G., & Förster, M. (2016, February). *Verhaltens- und Erlebensmuster von Lehramtsstudierenden unter dem Aspekt psychischer Gesundheit – eine stabile Beschreibung?* [Discussion Paper No. 23]. Europa-Universität Flensburg,

International Institute of Management. <https://www.uniflensburg.de/fileadmin/content/institute/iim/dokumente/forschung/discussion-papers/23-verhaltens-und-erlebensmuster-von-lehramts-studierenden.pdf>

Hamdan, W. (2012). *Psychische Gesundheit von Medizinstudierenden in Homburg/Saar*. [Doctoral Dissertation, University of Saarland]. https://publikationen.sulb.unisaarland.de/bitstream/20.
500.11880/21841/1/Hamdan_W_2012_Psychische_Gesundheit_von_Medizinstudierenden_in_Homburg.pdf

Jäger, T. (2017). *Instrumente des Human Resources Management unter besonderer Berücksichtigung von Copingstrategien im Rahmen von berufsbegleitenden Entwicklungsmaßnahmen.*

[Doctoral Dissertation, University of Sopron].

http://doktori.uni-sopron.hu/id/eprint/622/1/Disszert%C3%A1ci%C3%B3-Tina%20Jager.pdf

Kada, O. (2014). Wenn Studierende die gesundheitliche Situation Studierender erforschen. *Prävention und Gesundheitsförderung*, *9*(1), 22-28. https://doi.org/10.1007/s11553-013-0416-z

Kaub, K., Stoll, G., Biermann, A., Spinath, F. M., & Brünken, R. (2014). Interessenkongruenz, Belastungserleben und motivationale Orientierung bei Einstegern im Lehramtsstudium. *Zeitschrift für Arbeits- und Organisationspsychologie*, *58*(3), 125-139. <https://doi.org/10.1026/0932-4089/a000149>

Kötter, T., Kiehn, L., Obst, K. U., & Voltmer, E. (2021). The development of empathy and associated factors during medical education: A longitudinal study. *Journal of Medical Education and Curricular Development*, 8, 23821205211030176. <https://doi.org/10.1177/23821205211030176>

Künsting, J., Billich-Knapp, M., & Lipowsky, F. (2012). Profile der Anforderungs-bewältigung zu Beginn des Lehramtsstudiums. *Journal for Educational Research Online*, *4*, 84-119. https://www.pedocs.de/volltexte/2013/7482/pdf/JERO_2012_2_Kuensting_BillichKnapp_Lipowsky_Profile_der_Anforderungsbewaeltigung.pdf

Lüftenegger, M., Ning, C., Klug, J., & Spiel, C. (2019). *Berufsbezogene Risikomuster im Lehrerberuf* [White Paper]. <https://osf.io/p24q8>

Manderfeld, K., & Siller, H. S. (2018). Evaluation of an approach of professional role reflection in mathematics education. In B. Rott, G. Törner, J. Peters-Dasdemir, A. Möller (Eds.), *Views and beliefs in mathematics education* (pp. 103-113). Springer.

Martin, S. (2012). *Belastungserleben von Sportlehramtsstudierenden: Eine empirische Studie zur Messung der Belastungswahrnehmung.* GRIN Verlag.

Mašková, I., Mägdefrau, J., & Nohavová, A. (2022). Work-related coping behaviour and experience patterns, career choice motivation, and motivational regulation of first-year teacher education students – Evidence from Germany and the Czech Republic.

*Teaching and Teacher Education*, *109*, 103560. <https://doi.org/10.1016/j.tate.2021.103560>

Meier, S. (2015). *Kompetenzen von Lehrkräften: Eine empirische Studie zur Entwicklung fachübergreifender Kompetenzeinschätzungen.* Waxmann.

Meiseneder, K. (2015). *Burnout-Risiko von Lehramts- und Psychologiestudierenden in Österreich* [Master’s Thesis, University of Vienna].

<https://doi.org/10.25365/thesis.37256>

Nolle, T. (2013). *Psychosoziale Basiskompetenzen und Lernorientierung bei Lehramtsstudierenden in der Eingangsphase des Lehramtsstudium: Eine Untersuchung im Rahmen des Studienelements „Psychosoziale Basiskompetenzen für den Lehrerberuf“ an der Universität Kassel.* Julius Klinkhardt.

Nowik, D. & Franke, G. H. (2009). Stressbelastung von Studierenden moderner Bachelorstudiengänge – Erste Ergebnisse einer Pilotstudie an der Hochschule Magdeburg – Stendal (FH). *Proceedings of the 10th Nachwuchswissenschaftler-konferenz*, 140-147.

Nusseck, M., & Spahn, C. (2013). Vergleich der studienbezogenen Verhaltens- und Erlebensmuster bei Musikstudierenden des künstlerischen Hauptfaches und der Schulmusik. *Musikphysiologie und Musikmedizin*, *20*(3), 117-125.

Obst, K. U., & Kötter, T. (2020). Identifikation mit dem Studiengang als Ansatzpunkt für Resilienzförderung bei Studierenden. *Beiträge zur Hochschulforschung*, *42*(1-2),
148-161.

Obst, K. U., Voltmer, E., & Kötter, T. (2017). Identifikation mit dem Studiengang. *Prävention und Gesundheitsförderung*, *12*(4), 234-240. https://doi.org/10.1007/s11553-017-0596-z

Poterpin, E., Scheidl, G., & Ziegler, M. (2020, November 26). *Burnout-Risiko zukünftiger Lehrer*innen. Präventives Coachingangebot in der Ausbildung* [Poster Presentation]. Lehr-Lernprozesse gestalten, analysieren und evaluieren: 4. Jahrestagung zur Forschung an Pädagogischen Hochschulen im Verbund Nord-Ost, Vienna, Austria.

Reichl, C., Wach, F. S., Spinath, F. M., Brünken, R., & Karbach, J. (2014). Burnout risk among first-year teacher students: The roles of personality and motivation. *Journal of Vocational Behavior*, *85*, 85-92. <https://doi.org/10.1016/j.jvb.2014.05.002>

Ritter, J. C. (2017). *Stressbelastung bei Medizinstudierenden: Ist das Erlernen einer Entspannungstechnik während des vorklinischen Studiums sinnvoll und umsetzbar?* [Doctoral Dissertation, University of Lübeck].

https://www.zhb.uni-luebeck.de/epubs/ediss1929.pdf

Roloff Henoch, J., Klusmann, U., Lüdtke, O., & Trautwein, U. (2015). Die Entwicklung beruflicher Selbstregulation: Ein Vergleich zwischen angehenden Lehrkräften und anderen Studierenden. *Zeitschrift für Pädagogische Psychologie*, *29*(3-4), 151-162.

<https://doi.org/10.1024/1010-0652/a000157>

Römer, J., Appel, J., Drews, F., & Rauin, U. (2012). Burnout-Risiko von Lehramts-und Jurastudierenden der Anfangssemester. *Prävention und Gesundheitsförderung*, *7*(3), 203-208. https://doi.org/10.1007/s11553-012-0345-2

Römer, J., Drews, F., Rauin, U. & Fabricius, D. (2013). Riskante Studien- und Berufs relevante Merkmale von Studierenden: Ein Vergleich von Lehramts- und Jura-

studierenden. *Zeitschrift für Bildungsforschung, 3,* 153-173.

<https://doi.org/10.1007/s35834-013-0063-7>

Römer, J., Rothland, M., & König, J. (2017). Persönlichkeitsfaktoren und Kompetenzentwicklung in der Lehrerbildung: Zusammenhänge zwischen NEO-FFI, AVEM und Pädagogischem Wissen bei Lehramtstudierenden. *Psychologie in Erziehung und Unterricht*, *64*, 203-222. http://dx.doi.org/10.2378/peu2017.art13d

Rothland, M. (2011). Risikomerkmale von Lehramtsstudierenden. *Zeitschrift für Bildungsforschung*, *1*, 179-197. <https://doi.org/10.1007/s35834-011-0016-y>

Rothland, M. (2012). The professional motivation, job-related beliefs and patterns of work-related coping behaviour of teacher training students. In J. König (Ed.), *Teachers’ pedagogical beliefs. Definition and operationalisation – Connections to knowledge and performance – Development and change* (pp. 71-90). Waxmann.

Rothland, M. (2013). „Riskante“ Berufswahlmotive und Überzeugungen von Lehramtsstudierenden. *Erziehung und Unterricht*, *1-2*, 71-80.

Rothland, M., König, J., Darge, K., Lünnemann, M., & Tachtsoglou, S. (2014). Mit „männlicher Wucht“ in das „weibliche Biotop“? *Soziale Passagen*, *6*(1), 141-159. https://doi.org/10.1007/s12592-014-0155-9

Rumpler, M. (2013). *Burnoutrisiko bei österreichischen Lehramtsstudierenden in Zusammenhang mit studienrelevanten Merkmalen und der Wahrnehmung von*

*Belastungen*. [Master’s Thesis, University of Vienna]. https://doi.org/10.25365/thesis.29129

Schaarschmidt, U. (2005). Situationsanalyse. In U. Schaarschmidt (Ed.), *Halbtagsjobber? Psychische Gesundheit im Lehrerberuf – Analyse eines veränderungsbedürftigen* *Zustands* (pp. 41-71). Beltz.

Schaarschmidt, U. (2012). Eignung für den Lehrerberuf frühzeitig erkennen und kontinuierlich fördern. In B. Weyand, M. Justus, & M. Schratz (Eds.), *Auf den Lehrer/die Lehrerin kommt es an: Geeignete Lehrer/innen gewinnen, (aus-)bilden und fördern*, (pp. 60-79). Stifterverband.

Schaefer, C. (2012). *„Gestärkt für den Lehrerberuf“: Psychische Gesundheit durch Förderung berufsbezogener Kompetenzen.* [Doctoral Dissertation, University of Potsdam]. http://opus.kobv.de/ubp/volltexte/2013/6348/

Scholz, M., Neumann, C., Steinmann, C., Hammer, C., M., Schröder, A., Eßel, N., Paulsen, F., & Burger, P. H. M. (2014). Entwicklung und Zusammenhang von Arbeitsverhalten, Burnout-Beschwerden und Lebensqualität bei Studierenden der Humanmedizin vom Studienstart bis zum ersten Staatsexamen. *PPmP*, 65(3/4), 93-98. <http://dx.doi.org/10.1055/s-0034-1375630>

Schröder, E., & Kieschke, U. (2006). Bewältigungsmuster im Lehramtsstudium. Eine Untersuchung an den Universitäten Münster und Potsdam. In W. Schubarth & M. Pohlenz (Eds.), *Qualitätsentwicklung und Evaluation der Lehrerbildung. Die zweite Phase: Das Referendariat*(pp. 261-280). Universitätsverlag.

Störtländer, J. C., Fiedler-Ebke, W., Jürgens, E., Kindsvater, L., Klenner, D., Pieper, C., & Valdorf, N. (2020). Kritisch-reflexive Professionalisierung in schulischen Praxisphasen: Fünf Reflexionsangebote und ihre wissenschaftliche Begleitung im Rahmen des bildungswissenschaftlichen Lehramtsstudiums an der Universität Bielefeld. *Herausforderung Lehrer*innenbildung-Zeitschrift zur Konzeption, Gestaltung und Diskussion*, *3*(2), 399-435. <https://doi.org/10.4119/hlz-2538>

Voltmer, E., Bochmann, A., Kieschke, U., & Spahn, C. (2007). Studienbezogenes Erleben und Verhalten, Selbstaufmerksamkeit und soziale Unterstützung zu Beginn des Medizinstudiums. *Zeitschrift für medizinische Psychologie*, *16*(3), 127-134.

Voltmer, E., Kieschke, U., Schwappach, D. L., Wirsching, M., & Spahn, C. (2008). Psychosocial health risk factors and resources of medical students and physicians: A cross-sectional study. *BMC Medical Education*, *8*(1), 1-9. <https://doi.org/10.1186/1472-6920-8-46>

Voltmer, E., Kieschke, U., & Spahn, C. (2008). Studienbezogenes psychosoziales Verhalten und Erleben von Medizinstudenten im ersten und fünften Studienjahr. *Das Gesundheitswesen*, *70*(02), 98-104. https://doi.org/10.1055/s-2007-1022525

Voltmer, E., Köslich-Strumann, S., Voltmer, J. B., & Kötter, T. (2021). Stress and behavior patterns throughout medical education–a six year longitudinal study. *BMC Medical Education*, *21*(1), 1-12. <https://doi.org/10.1186/s12909-021-02862-x>

Voltmer, E., Köslich-Strumann, S., Walther, A., Kasem, M., Obst, K., & Kötter, T. (2021). The impact of the COVID-19 pandemic on stress, mental health and coping behavior in German university students–a longitudinal study before and after the onset of the pandemic. *BMC Public Health*, *21*(1), 1-15. <https://doi.org/10.1186/s12889-021-11295-6>

Voltmer, E., Kötter, T., & Spahn, C. (2012). Perceived medical school stress and the development of behavior and experience patterns in German medical students.

*Medical Teacher*, *34*(10), 840-847. <https://doi.org/10.3109/0142159X.2012.706339>

Voltmer, E., Obst, K., & Kötter, T. (2019). Study-related behavior patterns of medical students compared to students of science, technology, engineering and mathematics (STEM): a three-year longitudinal study. *BMC Medical Education*, *19*(1), 262.

https://doi.org/10.1186/s12909-019-1696-6

Voltmer, E., Rosta, J., Aasland, O. G., & Spahn, C. (2010). Study-related health and behavior patterns of medical students: A longitudinal study. *Medical Teacher*, *32*(10),
e422-e428. <https://doi.org/10.3109/0142159X.2010.496008>

Voltmer, E., Thomas, C., & Spahn, C. (2011). Psychosocial health and spirituality of theology students and pastors of the German Seventh-Day Adventist Church. *Review of Religious Research*, *52*(3), 290-305. <https://www.jstor.org/stable/23055553>

Wild, K., Scholz, M., Ropohl, A., Bräuer, L., Paulsen, F., & Burger, P. H. (2014). Strategies against burnout and anxiety in medical education–implementation and evaluation of a new course on relaxation techniques (Relacs) for medical students. *PloS One*, *9*(12), e114967. https://doi.org/10.1371/journal.pone.0114967

Wolf, S., Pfister, E. A., Schmicker, S., & Vajna, S. (2007). Gesundheitszustand und Gesundheitsförderung von Studierenden. *Praktische Arbeitsmedizin*, *9*, 6-11. http://www.bsafb.de/media/pa9_9_2007_gesundheitszustand_und_gesundheitsfoerderung_von_studierenden.pdf

Würfl, A. (2013). *Zusammenhang von Persönlichkeitseigenschaften und Risikomerkmalen bei Lehramtsstudierenden.* [Master’s Thesis, University of Vienna]. https://doi.org/ 10.25365/thesis.29346

Zehetner, G., & Boxhofer, E. (2018). Die WaLeKo-Studie. *Pädagogische Horizonte*, *2*,
23-46. <https://paedagogische-horizonte.at/index.php/ph/article/view/28/27>
